# Lotions of Sulphide of Potassium

**Published:** 1885-10

**Authors:** Pıerre Vigier


### Lotions of Sulphide of Potassium.

Sulphide of Potassium is a valuable remedy, but hitherto it could not be conveniently used on account of its disagreeable odor of putrid eggs, which, to most persons, is intolerable. The most certain method of dissolving it, and, at the same time, rendering it agreeable, is to add a quantity of tincture of benzoin equal to the amount of sulphide employed. Thus a solution composed of 15 grains of sulphide of potassium, 15 minims of tincture of benzoin and 3 ounces of water, emits a perfume much resembling the flower Acacia.—*Pierre Vigier, in Lyon Medical.*

---

*Le Journal d'Hygiene* gives the following comparative table, showing the probabilities of life for moderate drinkers and total abstainers:

| Moderate Drinkers. | | Total Abstainers. | |
|---|---|---|---|
| Age. | Expectation of Life. | Age. | Expectation of Life. |
| 20 | 15.6 | 20 | 44.2 |
| 30 | 13. | 30 | 36.5 |
| 40 | 11.6 | 40 | 28.8 |
| 50 | 10.8 | 50 | 21.5 |
| 60 | 8.9 | 60 | 15.285 |

It may be seen that, according to this table, the expectation of life is nearly two and one-half times greater for total abstainers than for those who are moderate drinkers.

---

Dr. B. W. Richardson has recently published a summary of the anæsthetics, from which we condense the following:

Considered safe: ethyl bromide, ethyl chloride, ether, ethene (olefiant gas), ethene chloride, methyl bromide, methyl chloride, methyl ether, methine chloride, methane (marsh gas), nitrous oxide.

Of doubtful value: amylene, amyl chloride, butyl chloride, benzene (benzol), carbon disulphide, carbon dioxide, carbon tetrachloride, methyl alcohol, methylal, spirits of turpentine.

Dangerous: amyl hydride, butyl hydride, carbon monoxide, ethyl hydride. Chloroform and ethene dichloride are regarded as useful, but requiring care. Dr. Richardson considers methylic ether to be the safest of all the anæsthetics.—*Polyclinic.*

